# Evaluating Mental Health Outcomes in COVID-19 ICU Survivors: A Scoping Review of Measurement Tools

**DOI:** 10.3390/jcm13113191

**Published:** 2024-05-29

**Authors:** Kimberly T’ng, Justin Kenardy, Andree Hartanto

**Affiliations:** 1 School of Psychology, University of Queensland, Brisbane, QLD 4072, Australia; j.kenardy@uq.edu.au; 2 School of Social Sciences, Singapore Management University, 10 Canning Rise, Level 5, Singapore 179873, Singapore; andreeh@smu.edu.sg

**Keywords:** mental health measures, ICU, critical illness

## Abstract

**Objective:** The objective of this scoping review was to map the range of measurement tools used to study the prevalence of common mental health conditions in COVID-19 ICU survivors. **Introduction:** Increased rates of admission to and survivorship from intensive care units (ICUs) have been observed in recent years, particularly during the global pandemic. ICU patients are at a higher risk of developing depressive, anxiety, and PTSD symptoms. Due to the high burden of disease, an accurate understanding of long-term mental health challenges for this population is key. Unfortunately, there is significant variability in reported prevalence rates. Heterogeneity in measurement tools potentially contribute to this. **Inclusion criteria:** Studies were eligible if they (a) reported mental health outcomes of adult patients diagnosed with COVID-19 and admitted to an ICU, (b) used standardised mental health outcome measures, and (3) were peer-reviewed. **Methods:** Searches were conducted in PubMed, PsycInfo, and Scopus. The initial search retrieved 1234 publications. After de-duplication and title and abstract screening, 72 full-text articles were examined for eligibility and 44 articles were excluded, leaving 28 eligible studies. Reference lists of the eligible studies were screened, and four other studies were added. 32 studies were ultimately included in this review. **Results:** Significant heterogeneity of measurement tools and clinical thresholds were observed. Only 6.25% of the studies compared changes in mental health outcomes to baseline measurements. Between five and nine unique measurement tools were used to study depression, anxiety, and PTSD, respectively. Studies were also observed to use up to 19 different thresholds to establish the prevalence of PTSD. **Conclusions:** The heterogeneity of measurement tools and thresholds continues to confound prevalence rate estimations of mental health complications post-ICU admission. Future research will benefit from consistency in the use of recommended outcome measures and the use of psychometrically comparable cut-off points between key measures.

## 1. Introduction

### 1.1. Post-Intensive Care Syndrome (PICS)

PICS is a syndrome characterised by new or worsened mental health, cognitive, or physical impairments after treatment in an intensive care unit (ICU) that can persist up to 15 years after discharge [1]. ICU survivorship can have a debilitating and lasting impact on patients in multiple domains of life. It is estimated that 50% of patients had a decreased ability to work and 25% needed assistance with activities of daily living 12 months after discharge from an ICU [2].

There is added reason to investigate PICS during the COVID-19 pandemic due to high rates of ICU admission and survival during the pandemic. In their systematic review and meta-analysis, Abate and colleagues [3] found that 32% of those infected with coronaviruses resulted in ICU admissions globally, and the prevalence rate of survival was 69%. COVID-19 ICU patients also tended to have medical complications including a longer ICU stay, the development of acute respiratory distress syndrome (ARDS) requiring sedation, and mechanical ventilation. Management of the global pandemic also required disease control measures, which potentially increased the risk of ICU survivors developing adverse psychological symptoms due to increased isolation (e.g., reduced visitation from family members) and reduced resources for psychosocial interventions [4]. Taken together, this means that there are now more critical illness survivors who have had ARDS but have had less psychosocial support during recovery and more environmental stressors than pre-pandemic patients.

### 1.2. Depression, Anxiety, and PTSD in ICU Survivors

The mental health domain of PICS includes symptoms of depression, anxiety, and post-traumatic stress disorder (PTSD) as they are the most common psychiatric complications after critical illness [1]. The experience of critical illness, or admission into an ICU, includes several stressors including invasive procedures, sleep deprivation, unnatural noise and light, difficulties communicating, and the threat of death due to critical illness [5]. Prior to COVID-19, pooled prevalence rates for mental disorders in ICU survivors were reported to be as high as 40% for anxiety [6], 34% for depression [7], and 34% for PTSD [8]. This is approximately ten times higher than the general population prevalence estimates [9,10].

The prevalence rate for mental health disorders might be even higher for ICU survivors with COVID-19. In total, 75% of COVID-19 ICU patients experience acute respiratory distress syndrome (ARDS) [11]. The hypoxemia and experience of dyspnoea (air hunger) during ARDS has been hypothesised to be psychologically traumatic for patients, contributing to post-ICU psychological sequelae [12]. Indeed, prior to the pandemic, depression, PTSD, and anxiety were, respectively, present in 36%, 39%, and 62% of ARDS ICU survivors [13]. Muysewinkel et al. [14] provided a comprehensive overview of PTSD studies during COVID-19 and found that a significant proportion of studies did not provide an index event for PTS symptoms and often used outdated DSM-IV criteria, potentially impairing the clinical relevance of the findings. It is thus likely that more COVID-19 ICU survivors are at risk of developing mental health complications post-ICU admission.

### 1.3. Prevalence Studies and Role of Outcome Measures

The prevalence of a disease indicates the number of people in a population that have a particular disease at a given point in time [15]. Having an accurate measure of prevalence rates is critical as it informs the magnitude of disease burden within a specific population. It serves to inform key community stakeholders on the development, prioritisation, delivery, and use of health services as well as their evaluation [16]. This need is particularly urgent, since depressive and anxiety disorders are the two most disabling mental disorders and are leading causes of the global health-related burden [17].

Unfortunately, there are large variances in reported prevalence rates of depression, anxiety, and PTSD within ICU survivors. Systematic reviews and meta-analyses indicate that prevalence rates ranged from 4% to 64% for depression [7], 5% to 73% for anxiety [6], and 4% to 62% for PTSD [8] in ICU survivors prior to COVID-19. Such variances have similarly been observed in research conducted on COVID-19 ICU survivors. For example, the prevalence rates for depression, anxiety, and PTSD a year after discharge were between 40% to 80% in one study [18] but 10% to 18% [19] in another. While COVID-19 has had a disproportionate impact on certain countries [20], variances in prevalence rates have also not been fully accounted for by geographical differences. Within the same country, depression was reported to be found in 37.8% [21] of patients in one study but 85.4% [22] in another study. This suggests that other factors might be at play.

The heterogeneity of measurements used between studies is one possible contributing factor to such variances. Systematic reviews conducted before COVID-19 reported significant heterogeneity in mental health outcome measures and cut-offs used for ICU survivors. Seven, four, and eight different measures were used for depression, anxiety, and PTSD, respectively, in three separate studies [6,7,8]). The I^2^ scores of pooled prevalence rates reported in the meta-analyses of those studies were also above 50%, indicating substantial heterogeneity [23]. All three reviews identified the heterogeneity in outcome measurement as a key inhibitor to a consolidated understanding of the prevalence rates of mental health concerns among ICU survivors. These findings were similarly echoed in the scoping review of ICU outcome measurements conducted by Turnbull and colleagues (2016) [24]. Despite being fairly dated, it is plausible that the heterogeneity in measurement tools used continues to limit this body of literature.

### 1.4. Research Gap

To the best of our knowledge, there have been no recent reviews focusing on outcome measures used to assess the mental health of COVID-19 ICU survivors. A preliminary search of the Cochrane Database of Systematic Reviews and PROSPERO was conducted on 5 April 2024 and no systematic reviews or scoping reviews on the topic were reportedly published or underway. A series of seminal reviews on the mental health outcomes of ICU survivors was also conducted approximately a decade ago between 2013 and 2015 [6,7,8,24]. Considering the long-lasting impact PICS can have and the mental health risks for COVID-19 ICU patients, an updated consolidation of prevalence rates and outcome measures used within the COVID-19 ICU population is needed.

A scoping review can be helpful to map the measurement tools used in research reporting the prevalence rates of mental health outcomes in COVID-19 ICU survivors. Compared to systematic reviews, scoping reviews are better suited to assess, understand, identify, map, and report the extent of the knowledge in an emerging field [25]. Within the context of this paper, a scoping review can be used to consolidate and map the tools used to measure mental health outcomes of COVID-19 ICU survivors and identify key research gaps so that future research efforts may be more strategically allocated.

### 1.5. Study Objective and Review Question

In an attempt to better understand the discrepancies in prevalence rates, this scoping review aims to (1) map out the mental health outcome measures used to measure the prevalence of mental health conditions in COVID-19 ICU survivors, and (2) preliminarily compare prevalence rates to those found before COVID-19. This study seeks to answer the following research question: How have differences in mental health assessment methods contributed to the variance in the prevalence of mental health conditions in COVID-19 ICU survivors?

## 2. Methods

### 2.1. Study Design

A scoping review was conducted in accordance with the Joanna Briggs Institute (JBI) methodology for scoping reviews [25]. Covidence was used to collate, manage, screen, and extract data from each study [26]. Microsoft Excel (Version 2208) was used to manage and organise the extracted data.

### 2.2. Search Strategy

**Figure 1:**
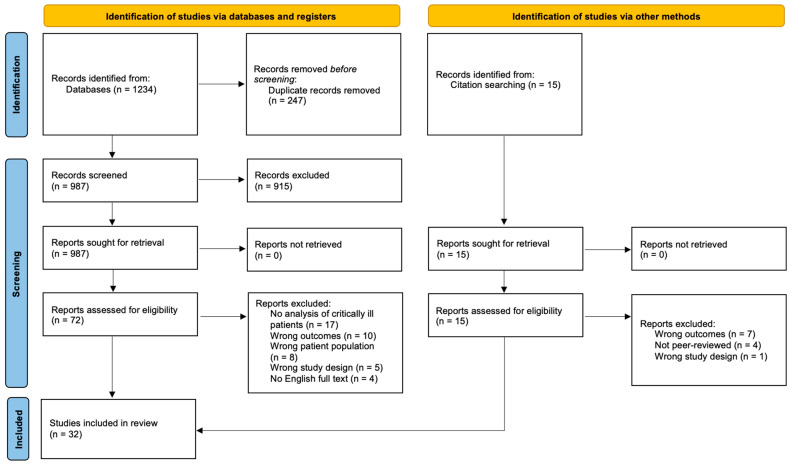
PRISMA flowchart.

The search strategy sought to locate published, English, peer-reviewed studies on the mental health outcomes of COVID-19 ICU survivors. A systematic search following the Preferred Reporting Items for Systematic Reviews and Meta-Analyses (PRISMA) 2009 flow diagram (Figure 1) was conducted on PubMed, PsycInfo, and Scopus on 1 July 2022. The specific search strategy for each database can be found in Appendix A.

The search terms across all databases included keywords related to COVID-19, ICU, survivors, and psychological distress. Where relevant, MeSH terms were used. Notably, although the search strategy was not limited by publication date, the nature of the topic meant that only articles published from 2020 were included. No contact with authors was initiated for further information on studies included.

### 2.3. Eligibility Criteria

Studies were eligible for inclusion if (1) the analysis included a full cohort or subgroup of critically ill adult patients with COVID-19, defined in our study as patients admitted to the ICU as a result of COVID-19, (2) mental health outcomes of ICU patients were reported, (3) study designs such as prospective and retrospective cohort studies, case–control studies, and cross-sectional studies were utilised, or (4) validated mental health measures were used.

Studies were excluded based on one or more of the following: (1) participants younger than 18 years and pregnant women; (2) no analysis of critically ill patients; (3) study designs that were qualitative, research letters, news reports, editorials, commentaries, case reports or case studies, recommendations, guidelines, or review articles; (4) non-English articles; (5) not peer-reviewed; (6) no full-text availability.

### 2.4. Study Selection

Following the search, all identified citations were collated and uploaded into Covidence [26] where duplicates were automatically omitted. The initial screening of titles and abstracts were completed by K.T. based on the outlined inclusion and exclusion criteria. Full-text screening was conducted by the same reviewer and reasons for exclusion were noted. Reference lists of eligible texts were also screened using the same process. Where there were areas of ambiguity, supervisor J.K. was consulted to confirm eligibility.

### 2.5. Data Extraction

Data extraction from papers included in the scoping review was completed by K.T. using a data extraction template developed by the reviewer. The data extracted included the following categories of relevant data: (1) lead author and year of publication; (2) country/region of the population studied; (3) study design; (4) sample size and demographic details; (5) outcome measures for depression, anxiety, and PTSD; (6) thresholds used; (7) prevalence of symptoms of depression/anxiety/PTSD; (8) key findings and implications of results.

The data extraction template was developed after the full-text eligibility of papers was determined. The extraction template focused only on assessment measures for depression, anxiety, and PTSD as these represented a comprehensive range of mental health challenges explored in the papers analysed. This review also focused on gaining an understanding of how the prevalence of mental health challenges has been studied. Data from the extraction process were then exported from Covidence to Microsoft Excel for further analysis. Authors of papers were not contacted for missing or additional data.

## 3. Results

Per JBI methodology, the PRISMA reporting guidelines were used in this segment [27]. The PRISMA flowchart in Figure 1 summarises the screening process performed for this scoping review [28]. The initial search retrieved 1234 publications. After the removal of 247 duplicates, 987 article titles and abstracts were screened according to a priori inclusion and exclusion criteria. Then, 915 articles were screened as irrelevant and full-text screening was undertaken on 72 studies.

In total, 44 articles were excluded at the full-text screening stage: 17 were excluded for lack of analysis on critically ill patients as defined by this review, 10 were excluded for a lack of target outcomes reported, eight for the wrong patient population (i.e., no analysis of COIVD-19 patients admitted to ICU), five for having an inappropriate study design, and four due to English full-text unavailability. Ultimately, 28 final studies were eligible for inclusion. Reference lists of the eligible studies were screened using the same process, and four other studies were assessed to be eligible. In total, 32 studies were included in this review.

### 3.1. Study Characteristics

Study characteristics have been summarised in Table 1. In total, the studies included 4318 participants, of which 1899 were admitted to the ICU. The total sample size in each of the 32 studies ranged from 9 to 675 participants. Of those participants, the ICU sample in each study ranged from 5 to 246 participants. The ICU samples predominantly consisted of male participants (*n* =1185). A detailed outline of the study characteristics may be found in Appendix B.

### 3.2. Use of Validated Measures

All but two studies used validated and standardised screening measures of the respective target outcomes [29,30]. Two studies incorporated the use of clinical interviews alongside the use of standardised screening measures [31,32].

### 3.3. Mental Health History, Baseline Measures, and Multiple Follow-Up Points

Less than half of the included studies included comparison groups in the study design or reported a history of psychiatric disorders within their sample (Table 2). Additionally, 19% (6/32) of studies were longitudinal and only 6.25% (2/32) of studies compared target outcomes with the mental health status at baseline.

### 3.4. Depression

#### 3.4.1. Measures

In total, 30 studies investigating depression used 5 different screening measures and 9 different thresholds to determine the presence of depression (Table 3). Additionally, 63% (19/30) of these studies used the Depression Subscale on the Hospital Anxiety and Depression Scale (HADS-D). While studies using the HADS-D varied slightly in thresholds, a majority (78%; 15/19) of these studies used the cut-off score of ≥8. The Patient Health Questionnaire (PHQ-9) was used in 26.67% (8/30) of studies reviewed, of which two used it in conjunction with a clinical interview. Studies using the PHQ-9 also varied in the thresholds used. Only 50% (4/8) used both ≥5 and ≥10 as thresholds for depression. 3.33% (1/30) used the Beck Depression Inventory, 3.33% (1/30) used the PROMIS-29, and 3.33% (1/30) used C19-YRS.

#### 3.4.2. Prevalence

A large variance was observed in the prevalence rates of depression within COVID-19 patients admitted to the ICU, ranging from 6.06% [33] to 85.41% [22]. For studies assessing depression using the HADS-D subscale, the difference in prevalence rates reported was at least 25% across all time points (Table 4). Notably, regardless of country or assessment time point, studies using the PHQ-9 tended to report prevalence rates of ≥15% (Table 5). The PHQ-9 was used in conjunction with clinical interviews in two studies, one reporting 11.76% prevalence rate of depression [31] and the other 59.18% [32].

### 3.5. Anxiety

#### 3.5.1. Measures

In total, 28 studies measuring anxiety used 5 different screening measures and 8 different thresholds to determine the presence of anxiety (Table 6). Additionally, 67.85% (19/28) of the studies included used the Anxiety Subscale on the Hospital Anxiety and Depression Scale (HADS-A). Moreover, 78.94% (15/19) of studies that used the HADS-A as a measurement included the cut-off score of ≥8. The General Anxiety Questionnaire (GAD-7) was used in 21.42% (6/28) of studies reviewed, of which one used it in conjunction with a clinical interview. Studies using the GAD-7 varied in the thresholds applied. Only 50% (3/6) used both ≥5 and ≥10 as thresholds for anxiety. The remaining 7.14% (2/28) used other measures of anxiety.

#### 3.5.2. Prevalence

A large variance was observed in the prevalence rates of anxiety within COVID-19 patients admitted to the ICU, ranging from 2.94% [34] to 62.50% [22] (Table 7). For studies using HADS-A, with eight as a cut-off score, it was observed that the lowest prevalence at each time band tended to be reported by studies from Netherlands and tended to be between 11.25% to 16.67% (Table 8).

### 3.6. PTSD

#### 3.6.1. Measures

In total, 22 studies measuring PTSD used 9 different measures with 19 different thresholds to determine the presence of PTSD. One study included the use of clinical interviews to identify PTSD. Additionally, 54% (12/22) of studies used the PCL-5, 27% (6/22) used the IES-R, 18% (4/22) used the IES-6, and 27% (6/22) used other PTSD measures. The IES-6 is a shortened version of the IES-R [45]. Notably, of these studies, two used different PTSD scales at different time points, but prevalence rates at each time point were not reported.

Studies that used the same PTSD screening measure also differed in the selected threshold. For PCL-5, the PTSD screening measure for most of the studies in this review, six different thresholds were used. Two studies using PCL-5 did not report the threshold used to determine prevalence. Five and three different thresholds were observed in studies using the IES-R and IES-6, respectively (Table 9). No two studies from the same country used the same measures and screening thresholds for PTSD.

#### 3.6.2. Prevalence

A large variance was observed in the prevalence rates of PTSD within COVID-19 patients admitted to the ICU, ranging from 5.88% [31] to 65.85% [46]. The variance in prevalence rates was 5.88% to 40% for the PCL-5 (Table 10), 9.76% to 33.33% for the IES-6 (Table 11), and 11.11% to 65.85% for the IES-R (Table 11). For studies that used mixed measures and other measures, the prevalence rate varied between 7.01% and 12.70% (Table 12) and 22.58% and 46.87% (Table 13), respectively.

## 4. Discussion

This review of 32 studies aimed to map out the mental health outcome measures used to monitor COVID-19 ICU survivors and preliminarily compare prevalence rates to those found before COVID-19. Overall, most of the included studies were conducted at a single centre and assessed all three target outcomes (i.e., depression, anxiety, and PTSD) at one time point up to 12 months after hospital or ICU discharge. This review demonstrates that a large range of measurement instruments continue to be used in assessing mental health outcomes among COVID-19 ICU survivors. Prevalence rates found in this review are similar in range compared to previous general ICU survivors. Other methodological limitations (e.g., limited comparisons to premorbid or baseline functioning) were observed in the included studies that further impeded a consolidated understanding of mental health complications in ICU patients.

Echoing previous reviews, this review demonstrates that the heterogeneity in outcome measures used to capture ICU outcomes remains a confounding factor within the extant literature. Five unique measurement tools were used to measure depression and anxiety, and nine were used to measure PTSD. Heterogeneity was also found in the clinical thresholds used. While the number of unique measures for each mental health outcome has reduced slightly since Turnbull and colleagues’ review [24], the distribution of studies using different measures has remained largely the same. Up to 67% of the studies included used the same measure and at least 20% of studies used another competing measure. In addition, this present review identified another layer of variance as multiple different clinical thresholds and cut-off scores were used for the same measures across studies. Muysewinkel et al. [14] highlighted that this heterogeneity is not only due to divisions in expert opinion but also cultural differences in the adoption of diagnostic criteria. Specifically, American studies generally use DSM-5 measures, while Asian studies often rely on DSM-IV criteria. This highlights the extent of heterogeneity in outcome measures that persists within the literature on ICU survivorship and the critical need for standardised and updated measures across different regions.

Recent efforts to remedy this situation reveal a continued schism. Expert groups have offered competing recommendations to use the HADS and IES-R [13] or shortened versions of the PHQ-9, GAD-7, and IES-R [55] to measure mental health outcomes of ICU survivors. The situation appears to be particularly problematic for the measurement of PTSD as there have been major developments in its criteria. Revisions made in the criteria for PTSD in the Diagnostic and Statistical Manual (DSM-5) led directly to the retirement of the IES-R by its developer [56] and the development of the PCL-5 [57]. However, experts within this area have written strident letters cautioning the move away from the IES-R or IES-6 towards the PCL-5 [56,58]. According to these experts, caution is necessary because the PCL-5 assesses the cluster of PTSD symptoms that is of unknown importance to critical illness survivors. Further, the PCL-5 has yet to be validated in the ICU survivor population, whereas the IES-R [59] and IES-6 [60] have. Together, it appears that the heterogeneity observed in this review is at least in part a reflection of divisions within expert opinion in the field.

Moving forward, research efforts should be directed towards evaluating the performance of outcome measures used and identifying comparable cut-off points. For instance, Snijkers and colleagues [61] contributed to diagnosis-specific guidelines by finding custom cut-off scores on the HADS that made it comparable to PHQ-9 and GAD-7 scores for patients with irritable bowel syndrome (IBS). Replicating such efforts for ICU survivors will allow future work to draw on existing data collected on survivorship outcomes. This will be particularly urgent considering the number of critical illness survivors following the COVID-19 pandemic.

Notably, other methodological limitations observed in this review might compound existing difficulties in consolidating outcome measures. Expert panels have had differing opinions on suitable outcome measures in part due to the lack of validated mental health outcome measures among ICU survivors. However, previous attempts to evaluate the psychometric performance of outcome measures for adult ICU survivors found that poorly conducted studies limited the evaluation of measurement quality [62]. A small sample size was a specific methodological limitation that was similarly observed in this review. While this is a natural limitation of this population, a sufficient sample size remains critical to robust findings. Therefore, future research efforts should also be directed towards collaboratively conducting multi-centre trials, which will improve both the rigour and sample size of studies on adult ICU survivors.

A secondary aim of this review was to preliminarily compare prevalence rates before and during the pandemic. Compared to prevalence rates prior to COVID-19, the prevalence rates found in this review fell within a similar range. Prior to COVID-19, prevalence rates ranged from 4% to 64% for depression [7], 5% to 73% for anxiety [30], and 4% to 62% for PTSD [8] in ICU survivors. In this review, the prevalence ranged from 6.06% [33] to 85.4% [22] for depression, 2.94% [34] to 62.50% [22] for anxiety, and 5.88% [31] to 65.85% [46] for PTSD among COVID-19 ICU survivors. This is interesting given the unique restrictions in hospitals and strain in resources that many healthcare systems have been under. Two possible hypotheses might account for this: (1) These additional contextual stressors might have had a lower than anticipated impact on ICU patients’ mental health, and (2) ARDS may have had less of an impact on the development of mental health sequalae as compared to other reasons for ICU admission (e.g., terminal illness or major surgery).

### 4.1. Limitations and Future Direction

#### 4.1.1. Within Studies

Studies in this review were predominantly in Western populations, conducted in single centres, with small sample sizes. This limits the rigour and generalisability of the findings. Studies in this review also had other methodological considerations that might affect the quality of their results. For instance, few studies within this review included an analysis of comparison groups, patients’ baseline measure of mental health, and patient history of psychiatric disorders. Notably, these methodological limitations have been cited in previous reviews. It appears that the field as a whole has had limited advancements. It is thus worth iterating that research efforts should focus on conducting multi-centre studies with a larger sample size to make findings in this body of literature more robust.

#### 4.1.2. Within Review

This review has several limitations. First, this review was conducted by a single rater, which limited the transparency and reliability of the review process. This was mitigated by consultation with a supervisor. Study authors were also not contacted for additional information on study results, which limited the findings to published results.

Second, this review was limited to studies utilising standardised measures of mental health outcomes. Several studies that were excluded often used health-related quality of life (HR-QoL) measures to estimate the prevalence of anxiety and depression. While such HR-QoL measures capture some evidence of mental health challenges, these measures are designed and validated to examine many aspects of a patient’s life rather than the distinct psychopathology of specific psychological conditions.

Lastly, it should be noted that studies in this review were conducted concurrently with the publication of expert group recommendations on ICU outcome measures, so this review might have mapped this body of literature on the cusp of consolidation.

## 5. Conclusions

Despite the increased volume of research in this space, the heterogeneity of measurement tools and thresholds continue to impede a consistent understanding of the development of mental health symptoms after ICU admission in patients with COVID-19. Future research efforts will benefit from coming to a unified agreement on recommended outcome measures, and from establishing psychometric comparability between key measures currently used to best leverage past research efforts.

## Figures and Tables

**Table 1:** Study characteristics.

Categories	*n* (%)
Country	
Austria	1 (3.13%)
Barcelona	1 (3.13%)
France	1 (3.13%)
Germany	1 (3.13%)
Ireland	1 (3.13%)
Italy	4 (12.50%)
Netherlands	6 (18.75%)
Sweden	1 (3.13%)
United Kingdom	2 (6.25%)
United States of America	6 (18.75%)
China	1 (3.13%)
Japan	1 (3.13%)
India	1 (3.13%)
Turkey	2 (6.25%)
Data collection period	
2020	27 (84.37%)
2020–2021	4 (12.50%)
2021	1 (3.13%)
Study design	
Prospective cohort	24 (75%)
Retrospective cohort	1 (3.13%)
Cross-sectional	5 (15.63%)
Case–series	2 (6.25%)
Single/multi-centre	
Single centre	24 (75%)
Multi-centre	8 (25%)
Target outcomes	
Depression, anxiety, and PTSD	20 (62.50%)
Depression and anxiety	8 (25%)
Depression only	1 (3.13%)
PTSD only	1 (3.13%)
Reported statistics	
Prevalence	26 (81.25%)
Median	16 (50%)
Mean, SD	6 (18.75%)
Assessment time point	
Single time point	26 (81.25%)
Two time points	5 (15.63%)
Three time points	1 (3.13%)
Studies at each follow-up time point	
Discharge up to 1 month	11 (34.37%)
1 months < x ≤ 3 months	12 (37.50%)
3 month < x ≤ 6 months	7 (21.87%)
6 months < x ≤ 1 year	4 (12.50%)

**Table 2:** Use of control groups and baseline measures.

Characteristic	Number of Studies (n/Total)
Included comparison groups in study design and analysis	13/32
Reported presence of history of psychiatric disorders	10/32
Provided details of psychiatric disorders	1/32
Compared target outcomes with baseline measure	2/32
Longitudinal (i.e., more than 2 follow-up points)	6/32

**Table 3:** Summary of measures and thresholds used to measure depression.

Threshold	Number of Studies
Beck Depression Inventory	
Not reported	1
C19-YRS	
Not reported	1
HADS-D	
≥8 borderline, ≥11 abnormal	1
≥8 mild, ≥11 moderate, ≥15 severe	2
HADS-D >10	1
HADS-D ≥10	1
HADS-D ≥8	12
Not reported	2
PHQ-9 and clinical interview	
PHQ-9 ≥10	2
PHQ-9	
Not reported	1
PHQ-9 > 10	1
≥5 mild, ≥10 moderate	1
0–4 minimal, 5–9 mild, 10–14 moderate, 15 or more severe	3
PROMIS-29	
Not reported	1
Total	30

**Table 4:** HADS-D ≥ 8: Lowest and highest prevalences of depression sorted by time.

Time Frame	HADS-D ≥ 8			
	Country; Reference	Lowest Prevalence (*n*/Total)	Country; Reference	Highest Prevalence (*n*/Total)
Discharge up to 1 month	Barcelona; Ojeda et al. [34]	7.69% (5/65)	Turkey; Kupeli et al. [35]	43.54% (27/62)
1 months < x ≤ 3 months	Netherlands; Vlake et al. [33]	6.06% (2/33)	Spain; Nanwani-Nanwani et al. [36]	20.96% (39/186)
3 month < x ≤ 6 months	Netherlands; Vlake et al. [37]	12.50% (10/80)	Japan; Banno et al. [38]	33.33% (6/18)
6 months < x ≤ 1 year	Netherlands; Heesakkers et al. [19]	18.29% (45/246)	Japan; Banno et al. [38]	44.44% (8/18)

**Table 5:** Prevalence and assessment time points for studies using PHQ9.

Reference	Country	Threshold	Time Point from Discharge/Diagnosis	Prevalence	Type	Severity
Kaur et al. [32]	India	PHQ 9 ≥10 and clinical interview	Discharge	59.18% (29/49)	Mean (SD)	10.5(3.3)
Liu et al. [39]	China	PHQ > 10	2 weeks	40% (14/35)	Not reported	Not reported
Martillo et al. [21]	United States	0–4 minimal, 5–9 mild, 10–14 moderate, 15 or more severe	1 month	38.63% (17/44)	Mean (SD)	5.7(6.9)
deGraaf et al. [31]	Netherlands	PHQ ≥10 and clinical interview	6 weeks	11.76% (4/34)	Not reported	Not reported
Vannorsdall et al. [22]	United States	≥5 Mild, ≥10 Moderate	4 months	85.41% (41/48)	Mean (SD)	6.7(4.6)
Daher et al. [40]	Germany	0–4 minimal, 5–9 mild, 10–14 moderate, 15 or more severe	6 months	Moderate—22.22% (4/18), Severe—5.55% (1/18)	Mean (SD)	6(5)
Cansel et al. [41]	Turkey	0–4 minimal, 5–9 mild, 10–14 moderate, 15 or more severe	2–8 months	Not reported	Mean (SD)	7.0(4.9)

**Table 6:** Summary of measures and thresholds used to measure anxiety.

Threshold	Number of Studies
C19-YRS	
Not reported	1
GAD-7	
GAD-7 > 10	1
0–4 minimal, 5–9 mild, 10–14 moderate, 15 or more severe	2
GAD-7 ≥ 5 mild, ≥10 moderate	1
Not reported	1
GAD-7 and clinical interview	
GAD-7 ≥ 10	1
HADS-A	
≥8 borderline or mild, ≥11 abnormal or moderate/severe	3
HADS-A > 10	1
HADS-A ≥ 10	1
HADS-A ≥ 8	12
Not reported	2
PROMIS-29	
Not reported	1
STAI	
Not reported	1
Total	28

**Table 7:** Prevalence and assessment time points for studies using GAD7.

Reference	Country	Threshold	Time Point from Discharge/Diagnosis	Prevalence	Type	Severity
Liu et al. [39]	China	GAD-7 > 10	2 weeks	17.14% (6/35)		
deGraaf et al. [31]	Netherlands	GAD-7 ≥10; clinical interview	6 weeks	2.94% (1/34)	Not reported	Not reported
Vannorsdall et al. [22]	United States	0–4 minimal, 5–9 mild, 10–14 moderate, 15 or more severe	4 months	62.50% (30/48)	Mean (SD)	5.1(4.9)
Daher et al. [40]	Germany	0–4 minimal, 5–9 mild, 10–14 moderate, 15 or more severe	6 months	Minimal to mild—89% (16/18) Moderate—5% (1/18), Severe—5% (1/18)	Mean (SD)	4(4)
Gilmartin et al. [42]	Ireland	Not reported	6 months	Not reported	Mean (SD)	5.8(6.1)
Cansel et al. [41]	Turkey	0–4 minimal, 5–9 mild, 10–14 moderate, 15 or more severe	2–8 months	Not reported	Mean (SD)	6.0(6.8)

**Table 8:** HADS-A: Lowest and highest prevalence of anxiety sorted by time.

Time Frame	HADS-A ≥ 8			
	Country; Reference	Lowest Prevalence (*n*/Total)	Country; Reference	Highest Prevalence (*n*/Total)
Discharge up to 1 month	Netherlands; Vlake et al. [33]	16.67% (5/30)	United Kingdom; MePeake et al. [43]	46.24% (43/93)
1 months < x ≤ 3 months	Netherlands; Vlake et al. [33]	15.15% (5/33)	Belgium; Rousseau et al. [44]	25% (8/32)
3 month < x ≤ 6 months	Netherlands; Vlake et al. [37]	11.25% (9/80)	Japan; Banno et al. [38]	44.44% (8/18)
6 months < x ≤ 1 year	Barcelona; Ojeda et al. [34]	10.76% (7/65)	United States; Rajajee et al. [18]	60% (6/10)

**Table 9:** Summary of measures and thresholds used to measure PTSD.

Threshold	Number of Studies
PCL-5	
1 re-experiencing, 1 avoidance, 2 negative alterations in cognition or mood symptoms, 2 arousal symptoms scored ≥2	1
PCL-5 ≥ 38	1
PCL-5 ≥ 31	3
PCL-5 > 33	2
PCL-5 ≥ 33	2
PCL-5 (short form) ≥ 8 (sum)	1
Not reported	2
IES-R	
IES-R > 1.6	1
IES-R > 33	1
IES-R ≥ 33	2
IES-R average ≥ 1.6	1
IES-R > 24 (sum)	1
IES-6	
IES-6 > 1.75 (average)	1
IES-6 ≥ 1.75	2
IES-6 > 10	1
TSQ	
TSQ ≥ 10 (sum)	1
PTSS	
PTSS-10 ≥ 35	1
PTSD Symptom Severity Scale	
1 re-experiencing, 2 increased activation, and 3 avoidance symptoms	1
PTSS14	
PTSS-14 >45	1
The National Stressful Events Survey PTSD Short Scale (NSESSS-PTSD)	
1 re-experiencing symptom, 1 avoidance symptom, 2 negative alterations in cognition or mood symptoms, and 2 arousal symptoms scored 2 (moderate) or higher	1
C19-YRS	
Not reported	1

**Table 10:** Prevalence of PTSD measured by PCL-5, sorted by assessment time point.

Reference	Country	Threshold	Time Point from Discharge/Diagnosis	Prevalence	Type	Severity
Liu et al. [39]	China	1 re-experiencing, 1 avoidance, 2 negative alterations in cognition or mood symptoms, 2 arousal symptoms scored ≥ 2	2 weeks	14.28% (5/35)	Not reported	Not reported
deGraaf et al. [31]	Netherlands	PCL-5 ≥ 38	6 weeks	5.88% (2/34)	Not reported	Not reported
Martillo et al. [21]	United States	PCL-5 ≥ 31	1 month	17.78% (8/45)	Mean (SD)	18 (21.2)
Monti et al. [47]	Italy	PCL-5; Threshold not reported	2 months	Not reported	Median (IQR)	7 (4–16)
Rass et al. [48]	United States	PCL-5 ≥ 33	3 months	12.90% (4/31)	Not reported	Not reported
Gilmartin et al. [42]	Ireland	PCL-5 ≥ 33	6 months	Not reported	Mean (SD)	21.1 (17.5)
Neville et al. [30]	Italy	PCL-5 (short form) ≥ 8 (sum)	6 months	19.70% (26/132)	Mean (SD)	2.48 (4.33)
Zangrillo et al. [49]	Italy	PCL-5; Threshold not reported	2 months	Not reported	Median (IQR)	7 (4–16)
			1 year	Not reported	Median (IQR)	7.5 (2–15)
Rajajee et al. [18]	Barcelona	PCL-5 ≥31	1 year	40% (4/10)	Median (IQR)	24 (17–37)

**Table 11:** Prevalence of PTSD measured by IES-R and IES-6, sorted by assessment time point.

Reference	Country	Threshold	Time Point from Discharge/Diagnosis	Prevalence	Type	Severity
Maley et al. [50]	United States	IES-6 > 1.75 (average)	Discharge	33.33% (20/60)	Not reported	Not reported
Carenzo et al. [46]	Italy	IES-R > 1.6	2 months	65.85% (27/41)	Mean (SD)	1.94 (0.75)
Rousseau et al. [44]	Belgium	IES-R ≥ 33	3 months	28.13% (9/32)	Median (IQR)	11 (4–24)
Vannorsdal et al. [22]	United States	IES-6 ≥ 1.75	4 months	25% (12/48)	Mean (SD)	1.2 (1.4)
Banno et al. [38]	Japan	IES-R average ≥ 1.6	4 months	11.11% (2/18)	Median (IQR)	7.5 (4.0–25.8)
			1 year	11.11% (2/18)	Median (IQR)	12.5 (2.0–27.5)
Vlake et al. [33]	Netherlands	IES-R > 24 (sum)	1 month	16.67% (5/30)	Median (IQR)	7 (1–51)
			3 months	18.18% (6/33)	Median (IQR)	8 (0–30)
Heesakkers et al. [19]	Netherlands	IES-6 ≥ 1.75	1 year	9.76% (24/246)	Median (IQR)	0.5 (0.2–1.2)

**Table 12:** Prevalence of PTSD measured by mixed tools, sorted by assessment time point.

Reference	Country	Threshold	Time Point from Discharge/Diagnosis	Prevalence	Type	Severity
Horn et al. [51]	France	IES-6 > 10 (T1) and PCL-5 > 33 (T2)	3 weeks	Not reported	Not reported	Not reported
			1 month after first evaluation	Not reported	Mean (SD)	9.6 (12.8)
ven den Borst et al. [52]	Netherlands	PCL-5 > 33 IES-R > 33	3 months	PCL-5: 10% (1/10) IES-R 0% (0/20)	Not reported	Not reported
Vlake et al. [37]	Netherlands	IES-R ≥ 33 (sum) TSQ ≥ 10 (sum)	6 weeks	7.01% (4/57)	Median (IQR)	9 (1–40)
			3 months	10.22% (9/88)	Median (IQR)	Not reported
			6 months	6.25% (5/80)	Not reported	Not reported
Weidman et al. [53]	United States	PTSS-10 ≥ 35, PCL-5 ≥ 31	20 days	12.70% (8/63)	Not reported	Not reported

**Table 13:** Prevalence of PTSD measured by other tools, sorted by assessment time point.

Reference	Country	Measure; Threshold	Time Point from Discharge/Diagnosis	Prevalence	Type	Severity
Nanwani-Nanwani et al. [36]	Spain	PTSD Symptom Severity Scale; 1 re-experiencing, 3 avoidance, 2 increased activation symptoms	3 months	22.58% (42/186)	Not reported	Not reported
Schandl et al. [54]	Sweden	PTSS-14; >45	5 months	34.78% (24/69)	Not reported	Not reported
Cansel et al. [41]	Turkey	The National Stressful Events Survey PTSD Short Scale (NSESSS-PTSD); 1 re-experiencing, 1 avoidance, 2 negative alterations in cognition or mood and 2 arousal symptoms scored 2 (moderate) or higher	2–8 months	Not reported	Mean (SD)	11.8 (14)
Halpin et al. [29]	United Kingdom	C19-YRS; Not reported	48 days	46.87% (15/32)	Not reported	Not reported

## Data Availability

The raw data supporting the conclusions of this article will be made available by the authors on request.

## Appendix A. Search Strategy

**Database**	**Search Strategy**	**Results**
PubMed	(coronavirus OR “corona virus” OR coronavirinae OR coronaviridae OR betacoronavirus OR covid19 OR “covid 19” OR nCoV OR “CoV 2” OR CoV2 OR sarscov2 OR 2019nCoV OR “novel CoV” OR “Coronavirus”[Mesh] OR “Coronavirus Infections”[Mesh] OR “COVID-19” [Supplementary Concept] OR “severe acute respiratory syndrome coronavirus 2” [Supplementary Concept] OR “Betacoronavirus”[Mesh]) AND (“critical illness”[MeSH Terms] OR “critical care”[MeSH Terms] OR “critical care”[tiab] OR “critical illness”[tiab] OR “intensive care”[tiab] OR “acute disease”[MeSH Terms] OR “acute disease”[tiab] OR “intensive therapy”[tiab] OR “high dependency”[tiab] OR Critically ill*[tiab]) AND (“depress*”[tiab] OR “anxi*”[tiab] OR “Post-traumatic stress” [tiab] OR “mental health conditions”[tiab] OR “psychological impact”[tiab] OR “Mental Disorders”[MeSH Terms] OR “psychology” [Subheading]) AND (“patient*” [tiab] OR “survivor*”[tiab] OR “Survivors”[Mesh] OR “inpatient*” [MeSH Terms])	664
Scopus	(TITLE-ABS-KEY((coronavirus OR “corona virus” OR coronavirinae OR coronaviridae OR betacoronavirus OR covid19 OR “covid 19” OR ncov OR “CoV 2” OR cov2 OR sarscov2 OR 2019ncov OR “novel CoV” OR “Coronavirus” OR “Coronavirus Infections” OR “COVID-19” OR “severe acute respiratory syndrome coronavirus 2” OR “Betacoronavirus”)) AND TITLE-ABS-KEY((“critical illness” OR “critical care” OR “critical care” OR “critical illness” OR “intensive care” OR “acute disease” OR “acute disease” OR “intensive therapy” OR “high dependency” OR critically AND ill*)) AND TITLE-ABS-KEY((“depress*” OR “anxi*” OR “Post-traumatic stress” OR “mental health conditions” OR “psychological impact” OR “Mental Disorders” OR “psychology”)) AND TITLE-ABS-KEY ((“patient*” OR “survivor*” OR “Survivors” OR “inpatient*”)))	454
PsycInfo	(Any Field: coronavirus OR Any Field: “corona virus” OR Any Field: coronavirinae OR Any Field: coronaviridae OR Any Field: betacoronavirus OR Any Field: covid19 OR Any Field: “covid 19” OR Any Field: nCoV OR Any Field: “CoV 2” OR Any Field: CoV2 OR Any Field: sarscov2 OR Any Field: 2019nCoV OR Any Field: “novel CoV” OR Any Field: Coronavirus OR Any Field: “Coronavirus Infections” OR Any Field: COVID-19 OR Any Field: “severe acute respiratory syndrome coronavirus 2” OR Any Field: Betacoronavirus) AND (Any Field: “critical illness” OR Any Field: “critical care” OR Any Field: “critical care” OR Any Field: “critical illness” OR Any Field: “intensive care” OR Any Field: “acute disease” OR Any Field: “acute disease” OR Any Field: “intensive therapy” OR Any Field: “high dependency” OR Any Field: “Critically ill*”) AND (Any Field: depress* OR Any Field: anxi* OR Any Field: “Post-traumatic stress” OR Any Field: “mental health conditions” OR Any Field: “psychological impact” OR Any Field: “Mental Disorders”) AND (Any Field: patient* OR Any Field: survivor* OR Any Field: Survivors OR Any Field: inpatient*)	116

## Appendix B. Study Characteristics

**Study ID**	**Country**	**Aim**	**Study Design**	**Data Collection Period**		**Year**	**Single/Multi Centre**	**History of Psychiatric Disorders Recorded**	**Total Sample (n=)**	**ICU Sample (n=)**	**Days in ICU (Mean, sd)**	**Age (Mean/Median)**	**Gender (% Male)**	**Comparison Group?**
				**Start**	**End**									
Banno et al., 2021 [38]	Japan	To assess the 1-year prevalence of post-intensive care syndrome after coronavirus disease 2019	Cohort study	Mar	Apr	2020	Single centre	No	18	18	6 (5.0–12.5)	57.5 (49.5–71.8)	78%	No
Cansel et al., 2021 [41]	Turkey	To investigate the prevalence of generalised anxiety disorder, depression, PTSD, and the factors that may be associated with them in patients who were hospitalised and then discharged due to COVID-19.	Cross sectional study	Mar	Nov	2020	Single centre	Yes	102	5	7.4 (5.1)	Not reported	Not reported	Yes
Carenzo et al., 2021 [46]	Italy	To describe the short-term HRQoL, physical function, and prevalence of post-traumatic stress symptoms of invasively mechanically ventilated COVID-19 patients treated in our urban tertiary academic ICU (more details on our ICU and hospital response to COVID can be found in reference 2).	Cohort study	Mar	May	2020	Single centre	No	47	47	15 (9–19)	59 (10)	79%	No
Daher et al., 2021 [40]	Germany	To investigate symptoms, abnormalities in pulmonary function, the prevalence of non-pulmonary organ dysfunctions and psychological disorders in patients who had been treated with IMV due to COVID-19 ARDS six months after discharge from hospital.	Cross sectional study	Feb	Apr	2020–2021	Single centre	No	18	18	34 (16)	61 (7)	61%	No
deGraaf et al., 2021 [31]	Netherlands	Potential sequelae of physiological and psychological impairment following SARS coronavirus variants warrant the need for a multidisciplinary evaluation of COVID-19 survivors to organise out-patient follow-up.	Cohort study	Mar	Jun	2020	Single centre	No	59	34	19.8 (12.5)	57.3 (11·1)	62%	Yes
Gilmartin et al., 2022 [42]	Ireland	To characterise the cognitive, psychological, and physical consequences of COVID-19 in patients admitted to the ICU and discharged alive.	Cohort study	Mar	Oct	2020	Single centre	No	22	22	21 (2–75)	52.4 (15)	68%	No
Halpin et al., 2021 [29]	United Kingdom	To inform service development, our multidisciplinary rehabilitation team examined the impact of COVID-19 on survivors discharged from hospital.	Cross sectional study	May	Jun	2020	Single centre	Yes	68	32	4	58.5 (34–84)	59%	Yes
Heesakkers et al., 2022 [19]	Netherlands	To assess the occurrence of physical, mental, and cognitive symptoms among patients with COVID-19 at 1 year after ICU treatment.	Cohort study	Mar	Jul	2020	Multi-centre	No	246	246	18.5 (11–32)	61.2 (9.3)	72%	No
Heyns et al., 2021 [63]	Belgium	To retrospectively analyse data obtained from the multi-domain assessment of hospitalised COVID-19 patients, to describe their health status at discharge, and to investigate whether subgroups of patients, more specific ICU patients and older adults (> 70 years), had more (or less) risk to experience specific impairments.	Cross sectional study	Apr	Jun	2020	Single centre	No	163	33	Not reported	60 (51.5–67.5)	Not reported	No
Horn et al., 2020 [51]	France	To assess the prevalence of and risk factors for post-traumatic stress disorder (PTSD) in patients with COVID-19.	Cohort study	Mar	May	2020	Single centre	Yes	180	55	8 (median)	Not reported	Not reported	Yes
Kaur et al., 2021 [32]	India	To estimate the prevalence of major depressive disorder (MDD) at two time points in individuals who have been hospitalised for the treatment of COVID 19.	Prevalence study	May	Oct	2020	Single centre	Yes	440	49	Not reported	Not reported	Not reported	Yes
Kupeli et al., 2021 [35]	Turkey	To investigate the degree of anxiety and depression in the first 24 h of people who were taken to the intensive care unit (ICU) due to COVID-19 and had to use unfamiliar devices in an unfamiliar environment.	Cohort study	Jan	May	2021	Single centre	No	62	62	N/A	57.1 (17.6)	68%	No
Liu et al., 2020 [39]	China	To report the prevalence of anxiety symptoms, depression symptoms, and PTSD for the hospital-discharged COVID-19 patients, and clarify risk factors for mental health problems among discharged COVID-19 patients.	Cross-sectional study	Jan	Apr	2020	Single centre	No	675	35	Not reported	Not reported	Not reported	No
Maley et al., 2022 [50]	United States	To determine the prevalence and extent of impairments impacting health-related quality of life among survivors of COVID-19 who required mechanical ventilation, 6 months after hospital discharge.	Cohort study	Mar	Dec	2020	Multi-centre	Yes	63	63	Not reported	59 (13)	46%	No
Martillo et al., 2021 [21]	United States	To determine the characteristics of post-intensive care syndrome in the cognitive, physical, and psychiatric domains in coronavirus disease 2019 ICU survivors.	Cohort study	Apr	Jul	2020	Single centre	Yes	41	41	10 (7–15)	53.9 (12.9)	80%	No
McPeake et al., 2021 [43]	United Kingdom	To understand the long-term psychosocial and physical consequences, including impact on employment, of severe COVID-19 infection, and explore if critically ill COVID-19 survivors have unique long-term outcomes, in relation to patients admitted to critical care without COVID-19.	Cohort study	Mar	May	2020	Multi-centre	Yes	299	93	11.1 (5–25.3)	59 (54–67)	66%	Yes
Monti et al., 2021 [47]	Italy	To assess the quality of life of invasively ventilated COVID-19 ARDS survivors.	Cohort study	Feb	Apr	2020	Single centre	No	39	39	10 (7–16)	56 (10.5)	90%	No
Nanwani-Nanwani et al., 2022 [36]	Spain	To determine the prevalence of PICS in a cohort of mechanically ventilated SARS- CoV-2 patients, assessed after 3 months of hospital discharge, in the ICU follow-up consultation facilities of three major hospitals in Spain.	Cohort study	Feb	May	2020–2021	Multi-centre	No	186	186	27 (14–56)	59 (12)	68%	No
Negrini et al., 2021 [64]	Italy	Report the cognitive and psychological features of the first consecutive patients with severe COVID-19 entering the post-acute phase, defined as clinical stability and complete weaning from sedative and antipsychotic drugs.	Case series	Mar	Apr	2020	Single centre	No	9	5	27 (21–29)	59 (59–64)	100%	No
Neville et al., 2022 [30]	United States	Examine long-term outcomes of patients who required intensive care unit (ICU) admission for severe COVID-19.	Cohort study	Mar	Dec	2020	Multi-centre	No	275	132	60 (2–13)	59.1 (47.5–68.8)	55%	No
Ojeda et al., 2022 [34]	Barcelona	Investigate the prevalence and characteristics of new- onset pain in COVID-19 ICU survivors 1 month after hospital discharge.	Cohort study	May	Jun	2020	Single centre	No	65	65	25 (15–33)	65 (57–70)	74%	Yes
Rajajee et al., 2021 [18]	United States	Prospectively describe 1-year outcomes, with a focus on functional outcome, cognitive outcome, and the burden of anxiety, depression, and post-traumatic stress disorder (PTSD) in COVID-19 patients managed with ECMO at our institution.	Prospective case series	Mar	Jul	2020	Single centre	No	23	14	51 (40–60)	41 (30–52)	57%	No
Rass et al., 2021 [48]	Austria	Quantify the impact of COVID-19 on mental health and health-related quality of life (QoL) 3 months after disease onset.	Cohort study	Apr	Sep	2020	Multi-centre	No	132	31	Not reported	58 (53–65)	77%	Yes
Rousseau et al., 2021 [44]	Belgium	Describe the mid-term outcomes and to assess the main PICS symptom prevalence in critically ill COVID-19 survivors referred to a face-to-face consultation in our post-ICU follow-up clinic at 3 months following a prolonged ICU stay.	Cohort study	Mar	Jul	2020	Single centre	No	32	32	23 (15–39)	62 (49–68)	72%	No
Schandl et al., 2021 [54]	Sweden	Evaluate long-term effects of COVID-19 in critically ill patients treated in ICU and whether invasive ventilation was associated with worse health-related quality of life and physical and psychological outcomes. To evaluate whether there were differences in lung capacity and function level at follow-up.	Cohort study	Mar	May	2020	Multi-centre	No	113	113	HFNo or NIV 4 (3–8) IMV 21 (15–30)	58 (12 and 14, respectively)	76%	No
Vannorsdall et al., 2022 [22]	United States	Prospectively characterise cognition, mental health symptoms, and functioning approximately 4 months after an initial diagnosis of COVID-19 in a racially and ethnically diverse group of patients.	Cohort study	Jul	Jan	2020–2021	Single centre	No	82	48	Not reported	58 (14.8)	48%	Yes
vanVeenendaal et al., 2021 [65]	Netherlands	Describe the physical, social and psychological impact on COVID-19 ICU survivors and their family members 3 and 6 months following ICU discharge.	Cohort study	Mar	Sep	2020	Single centre	No	60	60	19.4 (12.3–31.7)	62.5 (55.3–68)	68%	No
ven den Borst et al., 2021 [52]	Netherlands	Comprehensively assess these health domains in patients 3 months after recovery from acute COVID-19.	Cohort study	Apr	Jul	2020	Single centre	No	124	20	15 (8)	57 (10)	80%	Yes
Vlake et al., 2021 [37]	Netherlands	Quantify psychological distress up to three months after discharge in patients hospitalised during the first coronavirus disease 2019 (COVID-19) pandemic wave, assessed their HRQoL, explored predictors for psychological dis- tress and HRQoL, and examined whether psychological distress was more prevalent or more severe in COVID-19 confirmed patients, or in those treated in ICU.	Cohort study	Mar	Apr	2020	Single centre	Yes	294	40	16 (0–52)	62 (36–74)	68%	Yes
Vlake et al., 2021 [33]	Netherlands	To quantify short- and long-term psychologic distress, that is, symptoms of post-traumatic stress disorder, anxiety, and depression, and the health-related quality of life in coronavirus disease 2019 ICU survivors.	Cohort study	May	Apr	2020–2021	Multi-centre	No	238	118	10 (0–49)	61 (36–77)	67%	Yes
Weidman et al., 2022 [53]	United States	To report the prevalence of physical, psychological, and cognitive impairment among COVID-19 intensive care unit (ICU) survivors receiving follow-up care in an ICU recovery clinic, to assess for associations between PICS and ICU-related factors, and to compare the cohort of ICU survivors who attended a post-ICU clinic with a cohort of ICU survivors who did not.	Cohort study	Mar	May	2020	Single centre	No	87	87	22 (11–42)	62 (50–70)	74%	Yes
Zangrillo et al., 2022 [49]	Italy	1-year follow-up study of the same cohort to investigate long-term mortality, quality of life, functional and psychological recovery of the patients, as well as computed tomography (CT) characteristics of the lungs.	Cohort study	Feb	Apr	2020	Single centre	No	56	56	13 (9–21)	56 (11.9)	89%	No
